# Transcriptomic profiling of circulating tumor cells from metastatic breast cancer patients reveals new hints in their biological features and phenotypic heterogeneity

**DOI:** 10.1186/s40164-025-00659-y

**Published:** 2025-05-06

**Authors:** Tania Rossi, Sara Bandini, Michele Zanoni, Michela Cortesi, Michela Palleschi, Erika Bandini, Andrea Rocca, Giulia Gallerani, Ivan Vannini, Meropi Plousiou, Lorenzo Gerratana, Antonino Musolino, Giovanni Tallini, Giovanni Martinelli, Ugo De Giorgi, Paola Ulivi

**Affiliations:** 1 Biosciences Laboratory, IRCCS Istituto Romagnolo Per Lo Studio Dei Tumori (IRST) “Dino Amadori”, Meldola, Italy; 2 Medical Oncology, Breast & GYN Unit, IRCCS Istituto Romagnolo Per Lo Studio Dei Tumori (IRST) “Dino Amadori”, Meldola, Italy; 3 Department of Medical, Surgical and Health Sciences, University of Trieste, 34149 Trieste, Italy; 4 Department of Medical and Surgical Sciences (DIMEC), University of Bologna, 40138 Bologna, Italy; 5 Pathology Unit, Morgagni-Pierantoni Hospital, AUSL Romagna, Forlì, Italy; 6 Department of Medicine (DMED), University of Udine, 33100 Udine, Italy; 7 Department of Medical Oncology. CRO Aviano, National Cancer Institute, IRCCS, Aviano, Italy; 8 Solid Tumor Molecular Pathology Laboratory, IRCCS Azienda Ospedaliero-Universitaria Di Bologna, Bologna, Italy; 9 Department of Hematology and Sciences Oncology, Institute of Haematology “L. and A. Seràgnoli”, S. Orsola University Hospital, 40138 Bologna, Italy; 10 Department of Medical Oncology, IRCCS Istituto Romagnolo Per Lo Studio Dei Tumori (IRST) “Dino Amadori”, Meldola, Italy

**Keywords:** CTCs, Metastatic breast cancer, Gene expression analysis, RNA-sequencing, Metastasis, Liquid biopsy, DEPArray, Organotropism

## Abstract

The study of circulating tumor cells (CTCs) provides critical insights into the biological mechanisms underlying metastasis. This study aims to demonstrate the applicability of an integrated DEPArray-based phenotypic analysis combined with transcriptomic characterization to investigate the biology of CTCs isolated from 10 patients with metastatic breast cancer (MBC). The transcriptional profiles of CTCs were consistent with both the cancer type and epithelial characteristics. Gene set enrichment analysis identified pathways associated with synapse organization and calcium channel activity. Furthermore, distinct gene expression profiles were linked to specific metastatic sites, particularly bone metastases. We also report a rare and understudied population of CTCs, characterized by the co-expression of epithelial and leukocyte markers, observed exclusively in patient-derived samples and not in blood samples from healthy volunteers spiked with SKBR-3 and MCF-7 cell lines. This suggests that these double-positive CTCs (dpCTCs) may have a specific role in the metastatic process. The transcriptomic characterization of these rare CTCs enhances our understanding of their biology and potential involvement in metastasis. As a pilot study, our findings underscore the potential of CTC-based transcriptomics as a valuable tool for advancing clinical and biological understanding of MBC, particularly regarding metastatic mechanisms and organotropism. Moreover, it provides preliminary insights into dpCTCs, a poorly characterized population that may play a pivotal role in metastasis but remains largely unexplored. These findings could pave the way for developing targeted therapies aimed at the pathways driving metastasis and for improving patient monitoring through CTC profiling.

**Supplementary Information:**

The online version contains supplementary material available at 10.1186/s40164-025-00659-y.

To the editor,

Breast cancer (BC) is the most frequently diagnosed malignancy in women and one of the leading causes of cancer-related mortality worldwide. It is estimated that 20–30% of patients with early-stage BC eventually develop distant metastases, the primary drivers of mortality in these patients [1]. Despite advances, the mechanisms underlying metastasis remain incompletely understood, necessitating further investigation into the biological pathways involved. Circulating tumor cells (CTCs) play a pivotal role in the establishment of metastases and hold great promise as a non-invasive liquid biopsy tool [2, 3]. However, CTC investigation is challenging due to their rarity and heterogeneity, and the development of new workflows is required to achieve a comprehensive knowledge of CTC biology.

We previously described a workflow for phenotypic analysis using the DEPArray NxT platform (Menarini Silicon Biosystems), combined with single-cell RNA sequencing for gene expression profiling via the QIAseq UPX 3’ Transcriptome Kit (Qiagen) [4]. In this pilot study, we demonstrate the applicability of this workflow to CTCs isolated from patients with luminal MBC, aiming to elucidate their phenotypic heterogeneity and gene expression profiles (Fig. S1).

Prior to proceed with patient’s samples, feasibility experiments were conducted using SKBR-3 and MCF7 cell lines spiked in blood from healthy volunteers to test the antibody cocktail and the enriched detection rate (Fig. 1A, B).

**Fig. 1:**
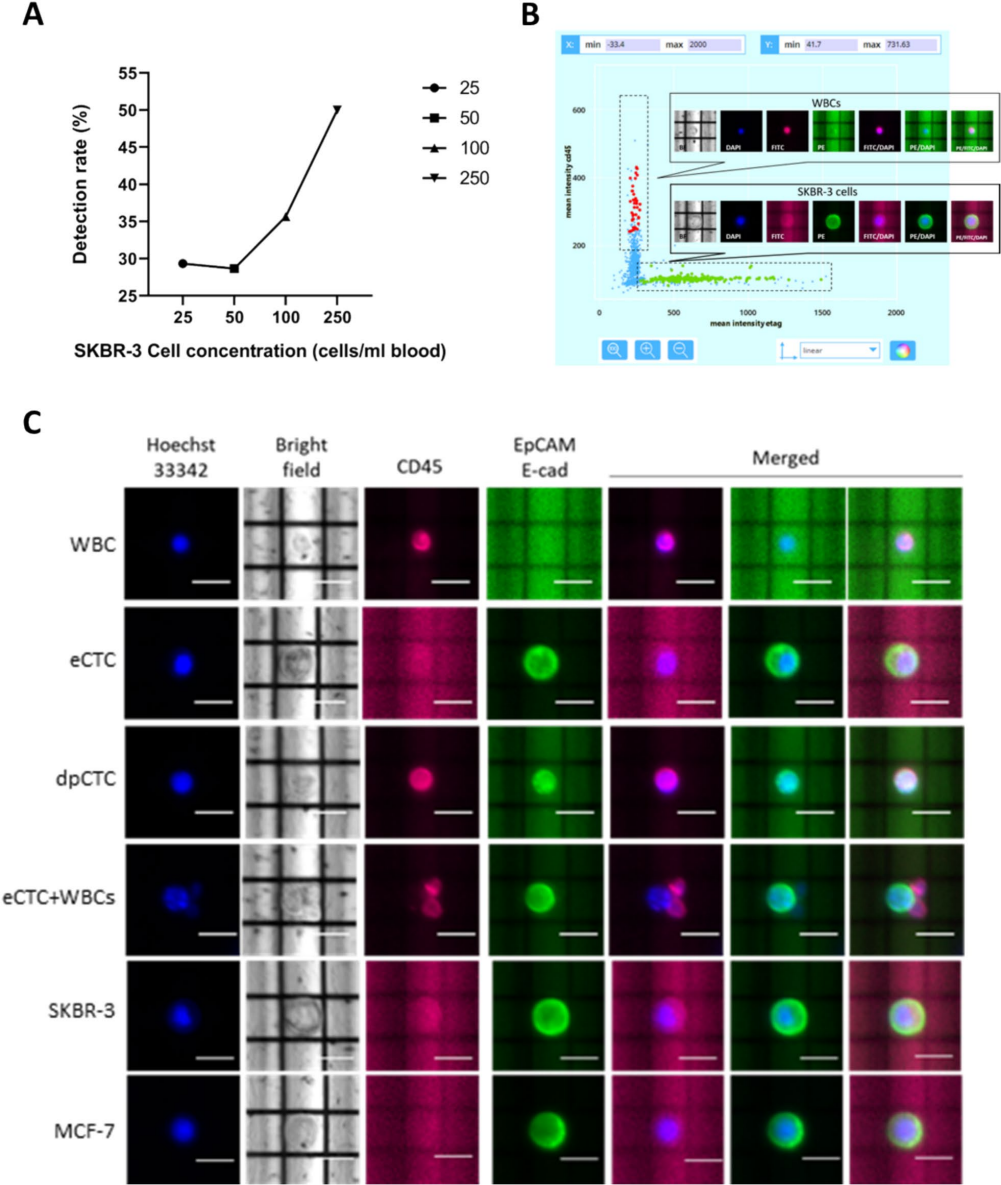
Experimental workflow set-up and phenotypic analyses.** A** Plot reporting the detection rate of the workflow (RosetteSep enrichment and DEPArray NxT detection) at each SKBR-3 cell concentration spiked in the peripheral blood of a healthy donor. In the x-axis, the concentration of each spike-in is reported (25, 50, 100, 250 cells/ml). In the *y*-axis the detection rate assessed with DEPArray NxT is reported for each spike-in. **B** Scatter plot created using the CellBrowser™ during DEPArray NxT analysis. The sample consists of 750 cells spiked in 3 ml of peripheral blood from a healthy donor at the concentration of 250 cells/ml. Enriched cells were marked with antibodies targeting EpCAM and E-cadherin (E-tag, PE channel, green fluorescence) and CD45 (FITC channel, purple fluorescence). DAPI channel was used for nuclear staining with Hoechst33342. A total of 375 SKBR-3 cells were detected (detection rate = 50%). On the x- and y-axes the mean intensity of PE and FITC channels are reported, respectively. SKBR-3 cells were detected as nucleus + /E-tag + /CD45- cells while WBCs were nucleus + /E-tag-/CD45 + . **C** Representative DEPArray images show different cell populations detected in patients and cell lines. WBCs are negative for epithelial markers (PE channel, green fluorescence), CD45 positive (FITC channel, purple fluorescence); CTCs shows epithelial markers positivity and were negative for CD45; CTCs with positivity for both epithelial markers and CD45, herein referred as double-positive CTCs (dpCTCs); heterotypic cluster composed by two WBCs and one epithelial CTC. Images of cell morphology are shown in the bright-field channel (grey), while cell nuclei are stained by Hoechst33342 (blue fluorescence). Scale bar: 30 µm

Validated antibody cocktail was used on viable CTCs, targeting the leukocyte marker CD45, and the epithelial markers EpCAM and E-cadherin (E-tag). Three distinct cellular populations were identified: leukocytes (CD45 + /E-tag-), CTCs (CD45-/E-tag +), and a minor subset of CTCs CD45 + /E-tag + , hereafter referred to as double-positive CTCs (dpCTCs). Interestingly, dpCTCs were absent in spike-in experiments with SKBR-3 and MCF7 cell lines in blood from healthy donors (Fig. 1C), suggesting a specific role in metastatic progression in MBC patients.

For gene expression analysis, CTCs isolated with DEPArray NxT were used for single-cell 3’RNAseq. Data were available for 74 CTCs from 10 MBC patients. To gain a comprehensive overview of CTC gene expression, we first grouped the reads from all the CTCs. Transcripts from 5,775 genes were detected (Table S1), among which transcripts associated with epithelial markers (e.g., E-cadherin, cytokeratins, EpCAM). GO enrichment by examining gene-disease association databases (DisGeNet, ClinVar2019, Human Phenotype Ontology) revealed that CTC expression profiles were consistent with BC (Table S2). Moreover, Gene Ontology (GO) enrichment analysis highlighted terms associated with calcium channel activity and the neural system (Fig. 2A), which have recently been linked to the metastatic potential of BC [5].

**Fig. 2:**
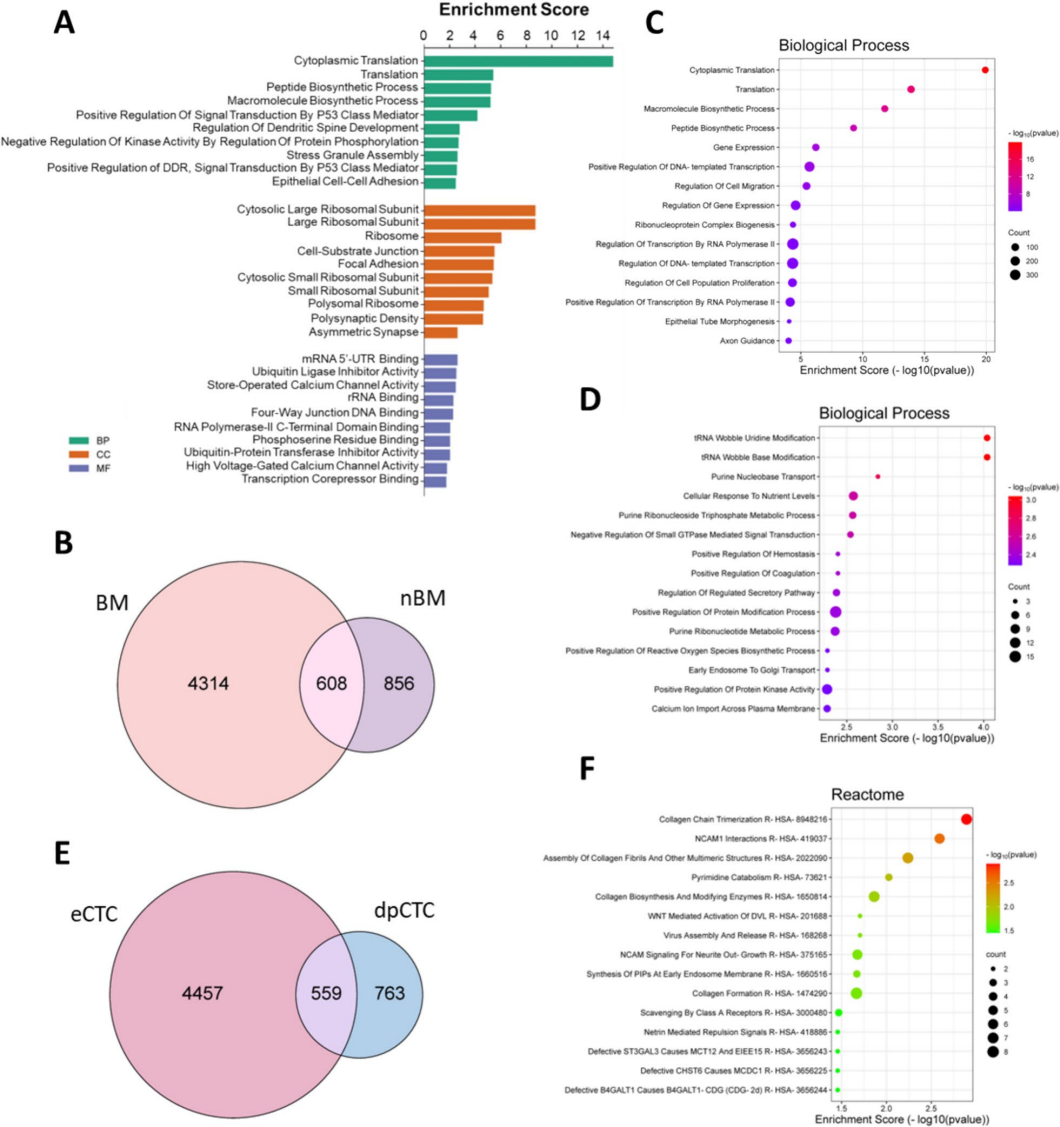
Transcriptomic profiling of CTCs. **A** Bar plot representing the 10 most enriched terms based on Gene Ontology (GO) Biological Process (BP), Cellular Component (CC) and Molecular Function (MF) in all the CTCs. **B** Venn diagram shows that globally 4,314 and 856 transcripts were privately detected in BM-CTCs and nBM-CTCs, respectively, while 608 genes were shared among the two groups. **C** Dot plots showing the 15 most significantly enriched terms using as input the list of the genes privately expressed by BM-CTCs and **D** nBM-CTCs. **E** Venn diagram shows that 4,457 and 763 were privately expressed by eCTCs and dpCTCs, respectively, while 559 transcripts were shared by the populations. **F** Bar plot of the 15 terms enriched with highest statistical significance based on the Reactome gene set library.

Given their role in organ-specific tropism [6], we hypothesized that CTCs exhibit distinct transcriptional signatures depending on the metastasis site. Accordingly, we analyzed the expression profiles and enriched terms of CTCs in relation to the specific metastatic sites. Grouped analyses were conducted on 54 CTCs from 8 patients MBC with bone metastasis (BM-CTCs). We detected a total of 4,922 transcripts (Table S3), while 1,464 transcripts were annotated in 20 CTCs isolated from 2 patients without skeletal metastasis (nBM-CTCs) (Table S4; Fig. 2B). In BM-CTCs, we detected genes known to be implicated in metastatic osteotropism, such as *HMGB1 *[7], *S100A4 *[8], and *VAPA *[9], and GO enrichment of private genes highlighted terms associated with cell migration, gene expression, axon guidance (Fig. 2C). A total of 4,314 genes were detected exclusively in BM-CTCs. To prioritize candidates potentially involved in BM, we focused on 42 genes expressed in at least 4 of the 8 bone-metastatic patients (Table S5). Gene enrichment analysis of this CTC-derived signature revealed biological processes associated with bone colonization and osteoclastogenesis, including response to lipopolysaccharide [10], autophagy [11] and blood vessel endothelial cell migration [12] (Fig. S2). *LIMK1*, a gene associated with peritoneal metastasis [13], was expressed by nBM-CTCs only. In this case, the GO enrichment of private genes resulted in enrichment of terms associated with tRNA Wobble Base modification (Fig. 2D).

Finally, we decided to better characterize dpCTCs. Grouped gene expression analysis on 12 dpCTCs from 3 mBC patients revealed the annotation of 1,322 transcripts (Table S6). By GO enrichment, we found that the most significantly enriched terms were primarily related to nervous systems and glycogen metabolism. Next, we compared the transcriptional profiles of dpCTCs to those of 62 epithelial CTCs (eCTCs) recovered from 7 MBC patients: 559 transcripts were expressed by both CTC populations, while 4,457 and 763 genes (Fig. 2E) were exclusively expressed by eCTCs and dpCTCs, respectively. GO enrichment of dpCTCs-specific genes revealed terms associated with collagen formation and NCAM1 interactions, based on the Reactome database (Fig. 2F).

The principal limitation of this study lies in the limited number of patients; a larger cohort of patients with nBM would have been required to perform a statistically robust comparison of gene expression profiles between CTCs from patients with BM and those with nBM. We were unable to increase the sample size due to the absence of detectable CTCs in some patients with nBM. This observation aligns with existing literature, which reports a higher prevalence of CTCs in BC patients with BM [14]. Furthermore, a high proportion of patients with luminal MBC present with bone involvement [15], which limits the possibility of expanding the cohort of luminal MBC patients without BM. Increasing the sample size would also facilitate the identification of a greater number of dpCTCs, a rare and poorly characterized population [16]. Notably, our study provides preliminary insights into dpCTCs, whose biological and clinical significance remains largely unexplored [16]. To our knowledge, this is the first study to report gene expression profiles specific to this CTC subpopulation. Another limitation of the study lies in the enrichment strategy, which is biased toward epithelial CTCs. Nevertheless, the workflow presented here—combining phenotypic identification and single-cell transcriptomic analysis—can be readily applied in future investigations targeting mesenchymal or hybrid CTC phenotypes, whose role in metastatic progression has been increasingly recognized [17].

In this pilot study, we demonstrate the feasibility of our workflow for studying CTCs from MBC patients, providing valuable insights into potential mechanisms underlying CTC biology, particularly in relation to metastatic sites and phenotypic characteristics. Data generated from our workflow support the notion that CTC transcriptional programs may encode organo-specific metastatic potential and provide gene expression signatures that correlate with the anatomical localization of metastases. These results emphasize the importance of extending this research to BC patients with different molecular subtypes to uncover signatures associated with additional metastatic sites (e.g., lung, liver, brain), thereby increasing statistical power and generalizability. Looking ahead, prospective studies involving patients with non-metastatic BC could be critical in validating organotropism-associated gene signatures in relation to clinically relevant endpoints, such as relapse-free survival and site-specific relapse. Despite the limitations inherent to a small cohort, our study establishes a solid foundation for future investigations aimed at improving personalized therapeutic strategies and advancing the clinical management of MBC.

## Supplementary Information

Additional file 1: Figure S1, Supplementary methods, Table S2, Table S5, Figure S2.

Additional file 2: Table S1. Transcripts detected by 3' RNA sequencing of circulating tumor cells (CTCs) isolated from 10 patients with luminal metastatic breast cancer. Reads from a total of 74 CTCs were analyzed in batch. The table lists all identified transcripts along with their normalized expression levels, reported in transcripts per million (TPM). 

Additional file 3:Table S3. Transcripts detected by 3' RNA sequencing of circulating tumor cells (CTCs) isolated from 8 patients with luminal metastatic breast cancer presenting bone involvement. Reads from a total of 54 CTCs were analyzed in batch. The table lists all identified transcripts along with their normalized expression levels, reported in transcripts per million (TPM).

Additional file 4:Table S4. Transcripts detected by 3' RNA sequencing of circulating tumor cells (CTCs) isolated from 2 patients with luminal metastatic breast cancer without bone involvement. Reads from a total of 20 CTCs were analyzed in batch. The table lists all identified transcripts along with their normalized expression levels, reported in transcripts per million (TPM).

Additional file 5: Table S6. Transcripts detected by 3' RNA sequencing of circulating tumor cells (CTCs) characterized by the phenotypic expression of epithelial (EpCAM, E-cadherin) and leukocyte (CD45) markers as assessed by DEPArray NxT platform, herein referred to as double-positive CTCs (dpCTCs). Reads from a total of 12 dpCTCs isolated from 3 patients with metastatic breast cancer were analyzed in batch. The table lists all identified transcripts along with their normalized expression levels, reported in transcripts per million (TPM).

## Data Availability

The datasets used and/or analyzed during the current study are available from the corresponding author on reasonable request.

## Abbreviations

CTC: Circulating tumor cell
MBC: Metastatic breast cancer
WBC: White blood cell
dpCTC: Double-positive CTC
eCTC: Epithelial CTC
GO: Gene ontology
BM: Bone metastasis
nBM: Non-bone metastasis
PE: Phycoerythrin
FITC: Fluorescein isothiocyanate
