# Supplementary material for: Transcriptomic profiling of circulating tumor cells from metastatic breast cancer patients reveals new hints in their biological features and phenotypic heterogeneity
Source: Exp Hematol Oncol. 2025 May 6;14:67. doi: 10.1186/s40164-025-00659-y (PMC12054220; doi:10.1186/s40164-025-00659-y)
Supplement: Supplementary file 1 — Additional file 1: Figure S1, Supplementary methods, Table S2, Table S5, Figure S2. [file 40164_2025_659_MOESM1_ESM.docx]

**Transcriptomic profiling of circulating tumor cells from metastatic breast cancer patients reveals new hints in their biological features and phenotypic heterogeneity**

*For a better understanding of our manuscript, we provide a graphical abstract, full material and methods section, and supplementary material.*

**Figure S1.** Graphical abstract summarizing the experimental approach and principal findings related to CTC heterogeneity and gene expression in metastatic breast cancer. Created with BioRender.com.


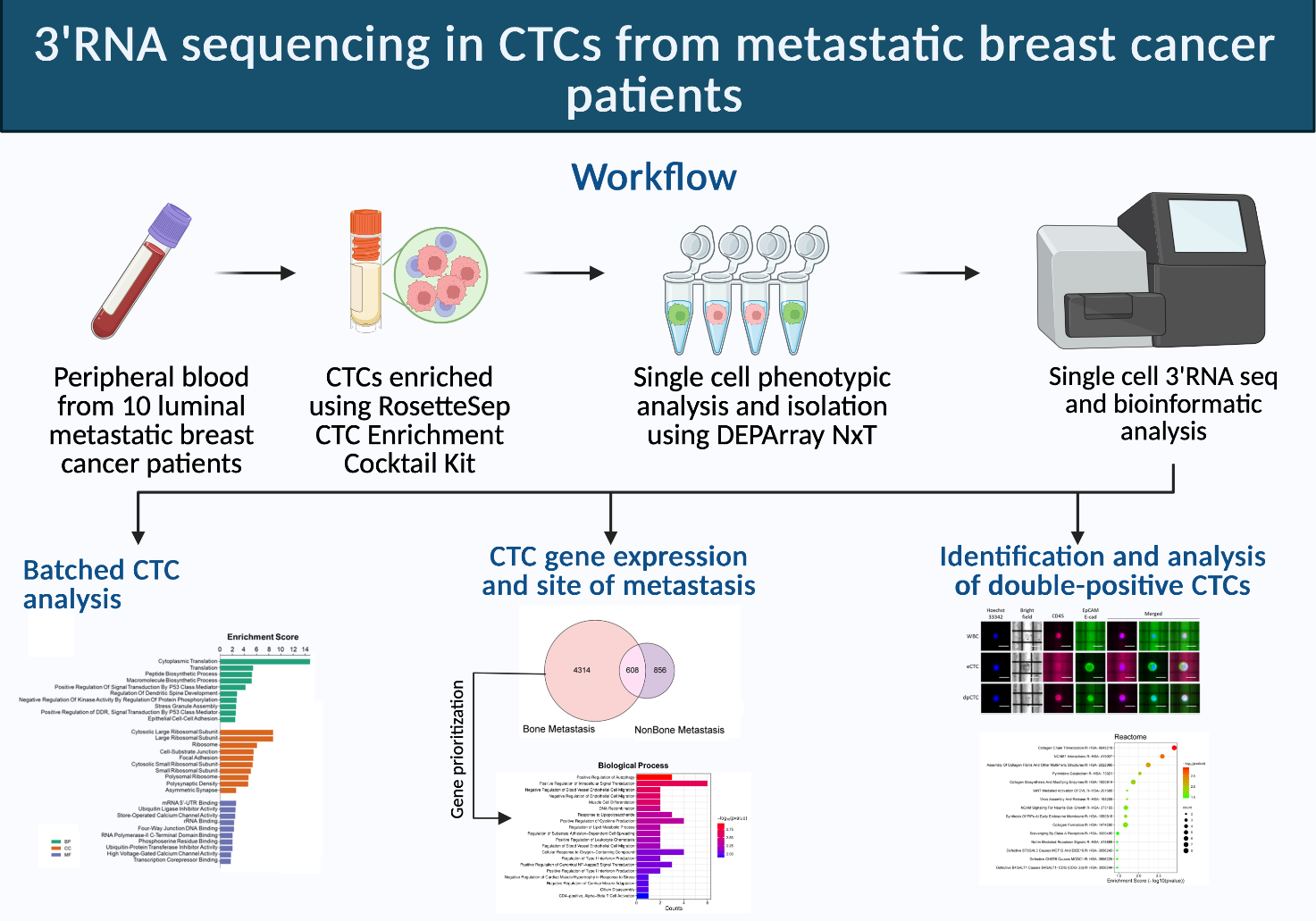


**Supplementary Methods**

**1. Patient samples**

Blood sample for circulating tumor cell (CTC) analysis were collected from patients with hormone receptor (HR)-positive (ER>10%), HER2-negative metastatic breast cancer (MBC). A prior history of non-breast malignancy (except for adequately controlled basal cell carcinoma of the skin, and other *in situ* carcinomas) was exclusion criterion. Approximately 7,5 ml of peripheral blood was collected from 10 patients with diagnosis of MBC in EDTA-tubes at different time points: before starting therapy, during treatment assessment (cycle duration 21 or 28 days) and every 4 cycles, and at end of therapy or time of disease progression (timepoint C). All the experiments were undertaken with the understanding and written consent of each subject. The present study has been approved by the local ethics committee (CEROM IRSTB174.19 and IRSTB114). Part of the patients were recruited in the KENDO phase II trial (NCT03227328). The study complied with the provisions of the Good Clinical Practice guidelines and the Declaration of Helsinki and local laws and fulfilled Regulation (EU) 2016/679 of the European Parliament and the Council of April 27, 2016, on the protection of natural persons regarding the processing of personal data.

**2. Cell Culture**

For set-up experiments, two breast cancer cell lines were used. SKBR-3 and MCF7 cell lines were obtained from ATCC (American Type Culture Collection) and maintained, respectively, in RPMI1640 medium (Euroclone, cat n. ECB9006L) and EMEM (ATCC, cat. n. 30-2003) supplemented with 10% FBS (Gibco™, Thermo Fisher Scientific, cat. n. A5256701), 2mM of L-glutamine (Gibco™, Thermo Fisher Scientific, cat. n. 25030024), 1% penicillin/streptomycin (Gibco™, Thermo Fisher Scientific, cat. n. 15140122), and 2% amphotericin B (Euroclone, cat. n. ECM0009D) in a humidified 37 °C, 5% CO2 incubator.

**3. Epithelial cell enrichment**

The enrichment of epithelial cells was performed using an immunodensity-based negative selection method, starting from peripheral blood of healthy volunteers spiked with cancer cell lines SKBR-3 and MCF7 for antibody cocktail validation experiments, and peripheral blood of MBC patients for CTC recovery. SKBR3 cell were used also spiked at different concentrations (250, 100, 50 and 25 cells/ml of blood) to 5 ml EDTA tubes containing ~3 ml of peripheral blood from a healthy donor to estimate the detection rate at the DEPArray. For cancer cell and CTC enrichment, 50 µl/ml of RosetteSep CTC enrichment cocktail containing anti-CD36 antibody (StemCell technologies, cat. n. 15167) was added directly to the blood-containing tube, mixed frequently and incubated at room temperature. After 20-minute incubation, processed blood was diluted with an equal volume of 1X PBS, and slowly poured on a SepMate tube (StemCell technologies, cat. n. 85450) together with 15 ml of Histopaque (Sigma-Aldrich, cat. n. 10771-500ML), and centrifuged 1200 x g for 10 minutes, brake on. The resulting cellular pellet was washed twice with 1X PBS and stored at -80°C in a freezing solution containing 90% FBS + 10% DMSO.

**4. Multi-antibody staining and DEPArray analysis**

Epithelial cell-enriched pellets from SKBR-3 and MCF7spiked sample, as well as from MBC patients, were subjected to antibody staining by mixing the following primary antibodies: anti-CD326 (EpCAM, clone HEA-125; 1:10 dilution; Miltenyi Biotech, cat. n. 130-113-264) and anti-CD324 (E-cadherin) (clone 67A4; 1:10 dilution; Miltenyi Biotech, cat. n. 130-128-815) to detect epithelial markers (E-tag; PE channel); anti-CD45 (clone HI30; 1:10 dilution; Invitrogen, cat. n. MHCD4520) to identify white blood cells (WBCs) (FITC channel). Nuclear staining was performed using Hoechst33342 (1 µg/ml; ThermoFisher Sc., cat. n. H3570) in the DAPI channel. After a 30-minute incubation at 4°C, the stained cellular pellet was washed twice with RPMI 1640 supplemented with FBS 10% and stored in the dark until use. For each sample, 12,5 µl of sample was loaded into a DEPArray Cartridge along with 2,5 ml of RPMI 1640 + FBS 10% and processed on the DEPArray NxT platform (Menarini Silicon Biosystems, Bologna, Italy). Samples were analyzed using the CTC-RUO live cells setting. Routability, defined as the percentage of cells that can be routed within the DEPArray cartridge compared to the number of identified cells, was evaluated as already reported in a previous publication from our group [1]. Cells of interest identified in samples from MBC patients were isolated as single cells in sterile 200 µl PCR-tubes, washed with 1X PBS and subjected to volume reduction, following the manufacturer’s guidelines for the DEPArray.

**5. RNA-sequencing and bioinformatic analysis**

For CTC gene expression analysis, libraries were prepared using the QIAseq UPX 3’ Transcriptome kit (Qiagen, cat. n. 333090) as reported in a previous paper from our group [1]. We included in library preparation the Xpress Ref Universal Total RNA (Qiagen, cat. n. 338112) (8 replicates) as a “control” sample. Briefly, immediately after being washed with 1X PBS, DEPArray-recovered CTCs were subjected to cell lysis using the reagents provided by the kit, and stored at -80°C until downstream analysis following the instructions provided in the protocol. During reverse transcription, each cell was given a different Cell-ID to be demultiplexed during bioinformatic analysis. Prepared libraries were quantified using the QIAseq Library Quantification Assay kit (Qiagen, cat. n. 333314) and Applied Biosystems 7500 Real-Time PCR system (ThermoFisher Sc.), while average fragment size was evaluated using the Agilent Bioanalyzer High Sensitivity. Lastly, libraries were combined in an equimolar pool, denatured with 0,1M NaOH and diluted to a final concentration of 3 pM. Paired end 100x27 sequencing was performed on V3-150 cycles cartridge using the Illumina MiSeq Sequencing system (Illumina Inc.). A custom sequencing primer was used as suggested by Qiagen, to save 23 bases of common sequence in the Read 2 adapter (Appendix C of the kit handbook).

Bioinformatic and statistical analyses were performed using the ready-to-use workflow (UPX 3’ RNA application) of the CLC Genomic Workbench 23.0.5 (Qiagen), Biomedical Genomics Analysis plugin. Fastq data were uploaded in the wizard tool as Illumina reads, and demultiplexed by selecting the specific Cell-ID used during reverse transcription. Gene expression tracks were generated through the “Quantify QIAseq UPX 3’” workflow with the following settings. For statistical analysis, the “Differential Expression for RNA-seq” workflow was used. Gene set enrichment analyses (GSEA) were performed using EnrichR web tool [2-4]. Enrichment and differential gene expression plots were prepared using SRplot [5].

**References**

1. Rossi T, Angeli D, Martinelli G, Fabbri F, Gallerani G. From phenotypical investigation to RNA-sequencing for gene expression analysis: A workflow for single and pooled rare cells. *Front Genet*. 2022;13:1012191. <https://www.ncbi.nlm.nih.gov/pubmed/36452152>. doi: 10.3389/fgene.2022.1012191.

2. Xie Z, Bailey A, Kuleshov MV, et al. Gene set knowledge discovery with enrichr. *Current protocols*. 2021;1(3):e90–n/a. <https://onlinelibrary.wiley.com/doi/abs/10.1002%2Fcpz1.90>. doi: 10.1002/cpz1.90.

3. Kuleshov MV, Jones MR, Rouillard AD, et al. Enrichr: A comprehensive gene set enrichment analysis web server 2016 update. *Nucleic acids research*. 2016;44(W1):W90–W97. <https://www.ncbi.nlm.nih.gov/pubmed/27141961>. doi: 10.1093/nar/gkw377.

4. Chen EY, Tan CM, Kou Y, et al. Enrichr: Interactive and collaborative HTML5 gene list enrichment analysis tool. *BMC Bioinformatics*. 2013;14(1):128–128. <https://www.ncbi.nlm.nih.gov/pubmed/23586463>. doi: 10.1186/1471-2105-14-128.

5. Tang D, Chen M, Huang X, et al. SRplot: A free online platform for data visualization and graphing. *PLOS ONE*. 2023;18(11):e0–e0294236. <https://www.proquest.com/docview/3069280650>. doi: 10.1371/journal.pone.0294236.

**Supplementary data**

| Database | Term | Overlap | P-value | Genes |
| --- | --- | --- | --- | --- |
| ClinVar2019 | Neoplasm of the breast | 22/27 | 2.13E-08 | *BARD1, XRCC3, PTEN, BRCA1, PHB, BRCA2, ESR1, PALB2, BRIP1, STK11, RAD51D, CASP8, RAD51, RAD51C, PIK3CA, CDH1, CHEK2, RB1CC1, AKT1, ATM, KRAS, TP53* |
| DisGeNET | Sporadic Breast Carcinoma | 65/159 | 7.54E-04 | *RB1, ACHE, CD40, THRA, OGG1, PTEN, BRCA1, COMT, NR3C1, BRCA2, CLDN1, IGF1R, PTBP1, STK11, CDH1, MYC, CHEK2, MYB, TNFSF10, SRRM2, PARP1, TSC1, ETV4, EMSY, TGFBR1, TGFBR2, MSH6, AR, RAD51C, MSH2, PIK3CA, MSH3, IRF1, PGR, TP53, RBMS3, MCPH1, DNMT1, CEBPD, PHB, FHIT, BRIP1, PMS1, BARD1, GADD45A, VDR, PNO1, CAV1, XRCC3, FANCA, IGF1, MLH1, ESR1, VEGFA, NFKBIA, GSTZ1, RAD51, TNFSF4, CDK2, BCL2, CD28, ATM, FGFR2, ZNF410, ATR* |
| Human Phenotype Ontology | Breast Carcinoma (HP:0003002) | 16/22 | 2.59E-05 | *PTEN, MLH1, ABCC11, STK11, BRIP1, MSH2, PIK3CA, CDH1, RB1CC1, CHEK2, CTNNB1, AKT1, KRAS, TP53, FGFR2, ATR* |

**Table S2.** List of terms associated based on EnrichR with breast cancer in circulating tumor cells (CTCs) based on their gene expression across the different gene set libraries queried.

**Table S5.** List of 42 genes identified as recurrently expressed in circulating tumor cells (CTCs) from patients with bone metastases. Each row corresponds to a gene, and each column represents a single patient. Binary values indicate gene expression status in CTCs from the respective patient: 1 = detected expression, 0 = not detected. Genes were selected based on expression in ≥4 out of 8 patients with bone metastases.

| Gene | P01 | P02 | P03 | P04 | P05 | P06 | P08 | P10 | Patients with transcript detected |
| --- | --- | --- | --- | --- | --- | --- | --- | --- | --- |
| *IGHMBP2* | 1 | 0 | 1 | 1 | 0 | 1 | 1 | 1 | 6 (75%) |
| *CADM1* | 1 | 1 | 1 | 1 | 0 | 1 | 1 | 0 | 6 (75%) |
| *KPNB1* | 1 | 0 | 1 | 1 | 0 | 1 | 1 | 1 | 6 (75%) |
| *FASTKD2* | 1 | 0 | 1 | 1 | 0 | 1 | 1 | 0 | 5 (62,5%) |
| *LOC105373943* | 1 | 0 | 1 | 1 | 0 | 1 | 1 | 0 | 5 (62,5%) |
| *ARHGEF26* | 1 | 1 | 1 | 0 | 0 | 1 | 1 | 0 | 5 (62,5%) |
| *LOC107986219* | 1 | 0 | 0 | 1 | 0 | 1 | 1 | 1 | 5 (62,5%) |
| *DBET* | 0 | 1 | 1 | 1 | 0 | 1 | 1 | 0 | 5 (62,5%) |
| *SYNC* | 1 | 0 | 0 | 1 | 0 | 1 | 1 | 0 | 4 (50%) |
| *PSMB2* | 1 | 0 | 1 | 0 | 0 | 0 | 1 | 1 | 4 (50%) |
| *NFIA* | 1 | 0 | 1 | 1 | 0 | 0 | 1 | 0 | 4 (50%) |
| *RGS16* | 0 | 0 | 0 | 1 | 1 | 1 | 1 | 0 | 4 (50%) |
| *ATP2B4* | 1 | 0 | 0 | 1 | 0 | 0 | 1 | 1 | 4 (50%) |
| *FLVCR1* | 1 | 0 | 1 | 0 | 0 | 1 | 1 | 0 | 4 (50%) |
| *BCL11A* | 1 | 0 | 1 | 1 | 0 | 1 | 0 | 0 | 4 (50%) |
| *LOC107986345* | 0 | 0 | 0 | 0 | 1 | 1 | 1 | 1 | 4 (50%) |
| *LOC105379094* | 1 | 0 | 1 | 1 | 0 | 0 | 1 | 0 | 4 (50%) |
| *HNRNPA0* | 0 | 1 | 0 | 1 | 0 | 1 | 1 | 0 | 4 (50%) |
| *NEDD9* | 1 | 0 | 1 | 0 | 1 | 0 | 1 | 0 | 4 (50%) |
| *TRIM56* | 1 | 0 | 0 | 1 | 1 | 0 | 1 | 0 | 4 (50%) |
| *DPP6* | 1 | 0 | 1 | 1 | 0 | 1 | 0 | 0 | 4 (50%) |
| *PLAG1* | 1 | 0 | 0 | 1 | 0 | 1 | 1 | 0 | 4 (50%) |
| *PRKCQ-AS1* | 1 | 0 | 0 | 1 | 0 | 1 | 1 | 0 | 4 (50%) |
| *SLC35E3* | 1 | 0 | 0 | 1 | 0 | 1 | 1 | 0 | 4 (50%) |
| *ANKRD20A19P* | 0 | 0 | 1 | 1 | 0 | 1 | 1 | 0 | 4 (50%) |
| *HMGB1* | 1 | 0 | 0 | 1 | 0 | 1 | 1 | 0 | 4 (50%) |
| *TRIM13* | 1 | 1 | 1 | 1 | 0 | 0 | 0 | 0 | 4 (50%) |
| *RDH11* | 1 | 0 | 1 | 1 | 0 | 1 | 0 | 0 | 4 (50%) |
| *RORA* | 1 | 0 | 1 | 1 | 0 | 1 | 0 | 0 | 4 (50%) |
| *LACTB* | 1 | 1 | 0 | 0 | 0 | 1 | 0 | 1 | 4 (50%) |
| *MYO9A* | 0 | 0 | 1 | 1 | 1 | 0 | 1 | 0 | 4 (50%) |
| *SNHG29* | 1 | 0 | 0 | 1 | 0 | 1 | 1 | 0 | 4 (50%) |
| *RPL27* | 1 | 0 | 0 | 0 | 0 | 1 | 1 | 1 | 4 (50%) |
| *BRIP1* | 1 | 0 | 0 | 0 | 0 | 1 | 1 | 1 | 4 (50%) |
| *NFIC* | 1 | 0 | 0 | 1 | 0 | 1 | 1 | 0 | 4 (50%) |
| *TICAM1* | 0 | 1 | 1 | 1 | 0 | 0 | 1 | 0 | 4 (50%) |
| *LOC105369202* | 1 | 0 | 1 | 0 | 0 | 1 | 1 | 0 | 4 (50%) |
| *ACTN4* | 0 | 1 | 1 | 1 | 0 | 0 | 1 | 0 | 4 (50%) |
| *PSMA7* | 1 | 1 | 0 | 0 | 0 | 1 | 1 | 0 | 4 (50%) |
| *SUPT20HL2* | 1 | 1 | 0 | 0 | 0 | 1 | 1 | 0 | 4 (50%) |
| *AMMECR1* | 1 | 0 | 0 | 0 | 1 | 1 | 1 | 0 | 4 (50%) |
| *INTS6L* | 0 | 0 | 1 | 1 | 1 | 0 | 1 | 0 | 4 (50%) |

**Figure S2.** Bar plot showing the top 20 most significantly enriched Gene Ontology – Biological Process (GO-BP) terms, based on the analysis of 42 genes exclusively expressed in circulating tumor cells (CTCs) isolated from bone metastasis patients. These genes were prioritized based on detection in at least 4 out of 8 patients.

**
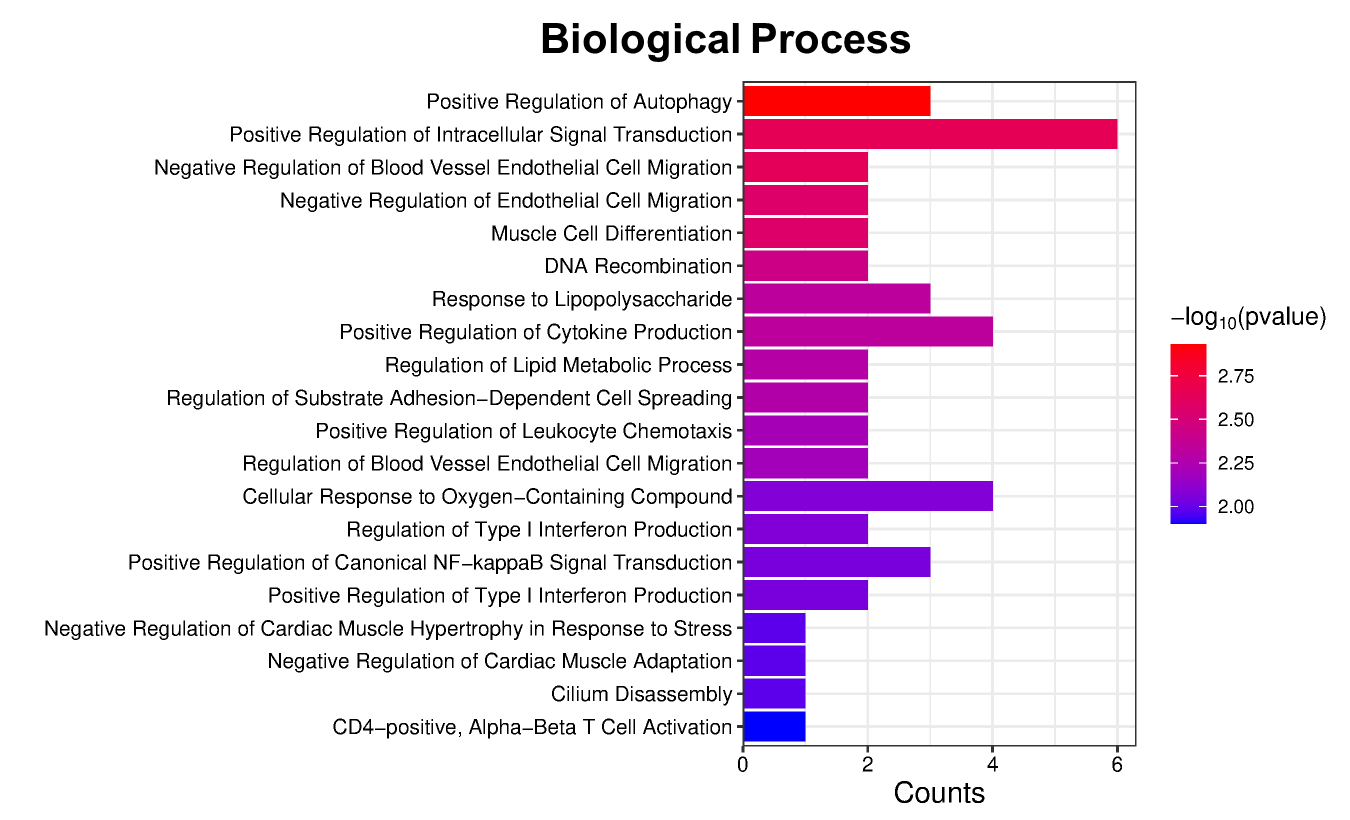
**
